# Nitrophenol Chemi-Sensor and Active Solar Photocatalyst Based on Spinel Hetaerolite Nanoparticles

**DOI:** 10.1371/journal.pone.0085290

**Published:** 2014-01-21

**Authors:** Sher Bahadar Khan, Mohammed M. Rahman, Kalsoom Akhtar, Abdullah M. Asiri, Malik Abdul Rub

**Affiliations:** 1 Center of Excellence for Advanced Materials Research (CEAMR), King Abdulaziz University, Jeddah, Saudi Arabia; 2 Chemistry Department, Faculty of Science, King Abdulaziz University, Jeddah, Saudi Arabia; 3 Division of Nano Sciences and Department of Chemistry, Ewha Womans University, Seoul, Korea; Queen's University Belfast, United Kingdom

## Abstract

In this contribution, a significant catalyst based on spinel ZnMn_2_O_4_ composite nanoparticles has been developed for electro-catalysis of nitrophenol and photo-catalysis of brilliant cresyl blue. ZnMn_2_O_4_ composite (hetaerolite) nanoparticles were prepared by easy low temperature hydrothermal procedure and structurally characterized by X-ray powder diffraction (XRD), field emission scanning electron microscopy (FESEM), X-ray photoelectron spectroscopy (XPS), Fourier transform infrared (FTIR) and UV-visible spectroscopy which illustrate that the prepared material is optical active and composed of well crystalline body-centered tetragonal nanoparticles with average size of ∼38±10 nm. Hetaerolite nanoparticles were applied for the advancement of a nitrophenol sensor which exhibited high sensitivity (1.500 µAcm^−2^ mM^−1^), stability, repeatability and lower limit of detection (20.0 µM) in short response time (10 sec). Moreover, hetaerolite nanoparticles executed high solar photo-catalytic degradation when applied to brilliant cresyl blue under visible light.

## Introduction

Environmental pollution has received extensive interest because of the uncertain consequences on human health and living organisms [1], [2]. Industrial activity causes many environmental problems by discharging vast quantities of toxic compounds into the environment. Many hazardous waste sites have been created by gathering pollutants in soil and water for many years [3], [4]. Therefore inspection of health risky pollutants in water and environment is an urgent demand for pollution controlling option. Thus easy, efficient and low cost processes for the recognition and detoxification of organic pollutants in aqueous solutions are needed to check and protect water resources and food supplies.

Several instrumental techniques exist for the recognition of organic pollutants, but are of less significance on the basis of efficiency and expense. However, sensor technology plays an important role in environmental safety and water treatment [5], [6]. Thus for environmental and health safety, it is essential to produce simple, reliable, and inexpensive sensors for the detection of p-nitrophenol (p-NP) in water, because nitrophenols are hazardous and toxic pollutants with inhibitory and biorefractory nature and have adverse effect on living organisms. Nitrophenols have been widely used in the manufacture of pesticides, dyes and pharmaceuticals. p-NP is a toxic derivative of the parathion insecticide and is carcinogenic, hazardous, mutagenic and toxic (cytotoxic and embryo-toxic) to mammals [7]. Due to high solubility and stability of p-NP in water, it has been found in freshwater, marine environments and has been detected in industrial wastewaters. Thus detection and monitoring of nitrophenols are crucial for environmental pollution control and industrial applications. Various chromatographic and spectroscopic techniques have been utilized for the detection and finding of hazardous solvents but their use is limited due to complication and sluggishness. Electrochemical sensors have achieved immense interest in the recognition and quantification of unsafe compounds since it is uncomplicated and rapid operation, response and recognition [8]. The sensitivity and selectivity of electrochemical sensor strongly dependent on the size, structure and properties of electrode materials and thus semiconductor nanostructured materials have received much importance and has extensively been utilized as a redox mediator in various sensors [9].

Photo-catalysis is also one of the low cost processes for detoxification of lethal organic compounds but the lack of active catalysts excluded this process from a wide range of applications. Among different photo-catalysts, TiO_2_ and ZnO have proven to act as dynamic photocatalysts and play pivotal roles in the detoxification of lethal organic compounds [10], [11]. However these photo-catalysts only encourage photo-catalysis upon irradiation by UV light because they absorb in the UV region. For solar photo-catalysis, a photo-catalyst must promote photo-catalysis by irradiation with visible light because solar spectrum consists 46% of visible light while the UV light is only 5–7% of the solar spectrum. Thus it is an urgent demand to develop an active photo-catalyst which can promote photo-catalysis in the visible region. Manganese oxides with various crystalline structures have been explored for various applications such as catalysis and electrode materials for lithium cathodes [12], [13]. However, to enhance the various properties of manganese oxide to meet the increasing needs for different applications, the features of manganese oxide must be modified. One of the main noteworthy methods to amend the characteristics of these nanomaterials is the introduction of doped materials in the parent system because recently doped metal oxides have shown excellent properties in various applications [14].

Therefore in this study, spinel (AB_2_O_4_) like zinc doped manganese oxide (spinel hetaerolite) has been synthesized by a simple low-temperature hydrothermal process and characterized by XRD, FESEM, XPS, FTIR, XPS and UV-vis. spectroscopy. From an application point of view, spinel hetaerolite was investigated as a sensor substance for the discovery of p-NP and solar photo-catalyst for the degradation of brilliant cresyl blue under visible light. To the best of our knowledge, this is the first detail of a spinel heterolite as a nitrophenol sensor and solar photo-catalyst for brilliant cresyl blue.

## Methods

### 1. Synthesis of hetaerolite nanoparticles

An equi-molar aqueous solution was prepared by dissolving 1.36 g of ZnCl_2_ and MnCl_2_.4H_2_O in 100 ml distilled water and further titrated by NH_4_OH solution to increase the pH above 10.0. This high basic solution was then shifted to a Teflon autoclave and kept at 150.0°C for 16 hours in an oven. Finally a black product was obtained by washing the precipitate. The precipitate was dried and calcined at 400.0°C for 5 hours.

### 2. Possible growth of hetaerolite nano-particles

**Figure 1:**
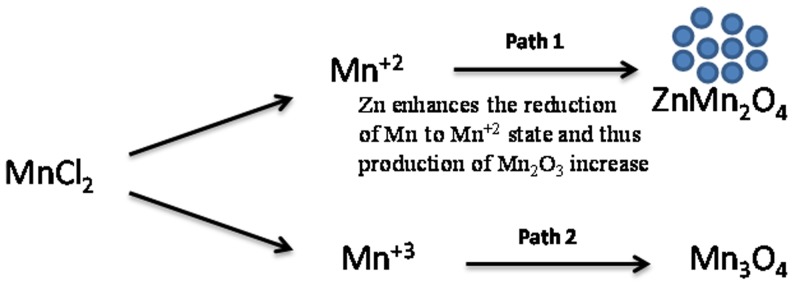
Possible growth mchanism of hetaerolite nanoparticles.

In the case of manganese oxides formation, it has been reported that manganese gets reduced to Mn^3+^ or Mn^2+^ states and generates lamellae structure which then appear in the form of rod like structure to reduce the surface energy [15]–[17]. After hydrothermal process it crystallizes to form MnO-OH nanorods. The calcination of MnO-OH gives Mn_3_O_4_ due to dehydration and oxygen out diffusion at high temperature [18]. However the incorporation of a Zn precursor has great influence on the reduction of manganese. It enhances the reduction process of Mn by first reducing to Mn^+3^ state and further reduce Mn^+3^ to Mn^+2^ state which result in the formation of ZnMn_2_O_4_ nanoparticles. Schematically probable growth process of ZnMn_2_O_4_ nanoparticles is given in Figure 1.

### 3. Characterization

JEOL Scanning Electron Microscope (JSM-7600F, Japan) was used to analyze the morphology of the prepared material while the crystallography was studied by X'Pert Explorer, PANalytical X-ray diffractometer. The XPS spectrum was recorded in the range of 0 to 1350 eV by using a Thermo Scientific K-Alpha KA1066 spectrometer (Germany). For compositional, optical and degradation studies of the nanomaterial, FT-IR and UV spectrum were record by a PerkinElmer (spectrum 100) FT-IR spectrometer and PerkinElmer (Lambda 950) UV-visible spectrometer. For sensing study, I–V measurements were carried out using an electrometer (Kethley, USA).

### 4. Fabrication of chemical sensor

Hetaerolite nanoparticles were coated as a thin film on the surface of a glassy carbon electrode (GCE, surface area 0.0314 cm^2^) and dried at 60.0°C for 12 hours. Time delaying and response time were 5.0 sec and 10.0 sec, respectively. The sensing ability of the hetaerolite nanoparticle modified GCE was evaluated using nitrophenol in a similar way as we published earlier [19], [20].

### 5. Solar photo-catalytic degradation

Solar photo-catalytic activity of hetaerolite nanoparticles was evaluated through degradation of brilliant cresyl blue under sun light. The dye is stable under visible light irradiation in the absence of a photo-catalyst. In photo-catalytic degradation, two different 100.0 mL, 1×10^−4^ M of brilliant cresyl blue solutions were taken in different beakers and adjusted the pH to 5, 8 and 10, by drop wise addition of 0.2 M NaOH solution under vigorous stirring. 0.1193 g and 0.1132 g catalyst were then added into the dye reaction solutions. The solution was then irradiated under sunlight at constant stirring and 4–5 mL of dye solutions were pipetted out at regular interval and measured the absorbance at λ_max_ = 595.0 nm using UV visible spectrophotometer. Dye degradation without catalyst was also studied under visible light to check any degradation of dye.

## Results and Discussion

### 1. Physiochemical characterization of hetaerolite nanoparticles

**Figure 2:**
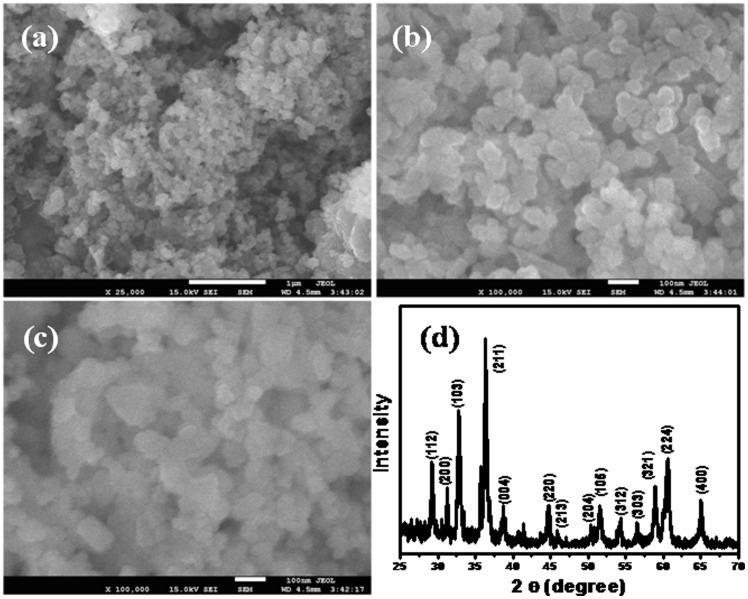
Typical low and high-resolution FESEM images (a–c) and XRD pattern (d) of the synthesized hetaerolite nano-particles.

FESEM was utilized to explore the morphology and size of hetaerolite. The morphology of hetaerolite was illustrated by FESEM which are shown in Figure 2 (a–c). It is evident from the FESEM images that the synthesized material is composed of particles having average diameter of ∼38±10 nm. These nanoparticles were prepared in large quantity possessing a spherical shape.

The crystallographic information of the hetaerolite nano-particles were corroborated by X-ray diffraction (Figure 2 (d)). All the characteristic diffraction peaks coincided with those for well-crystalline distinct body-centered tetragonally structured of ZnMn_2_O_4_. X-ray diffraction peaks of the samples are in outstanding agreement and excellent accordance with the JCPDS card no. 077-0470 [21]. According to the JCPDS card, the synthesized product is a body-centered tetragonal phase ZnMn_2_O_4_ with cell parameters of a = 5.72 Å and c = 9.24 Å and space group of 141/amd(141). All diffraction peaks were only related to ZnMn_2_O_4_ without any impurity peaks and thus the synthesized product therefore consist of pure ZnMn_2_O_4_ crystals. The size of hetaerolite nano-particles (38 nm) suggested by FESEM was also verified and supported by Scherrer formula.
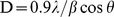
Where λ is the wavelength of X-ray radiation, β is the full width at half maximum (FWHM) of the peaks at the diffracting angle **θ**.

**Figure 3:**
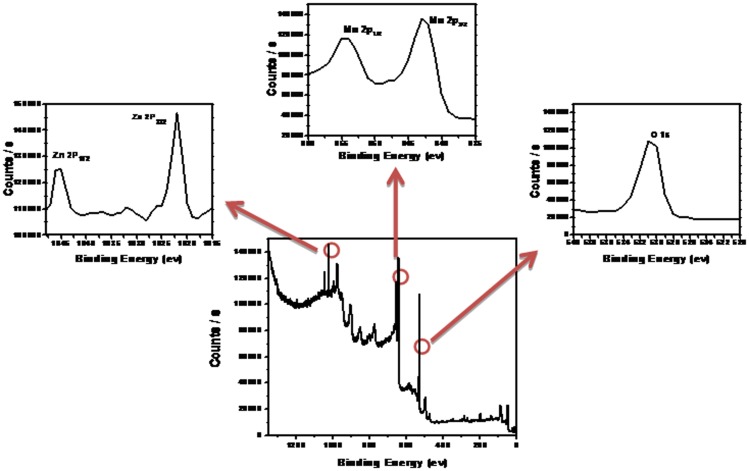
Typical XPS spectrum of the synthesized hetaerolite nano-particles.

XPS was analyzed to study the composition of hetaerolite nano-particles and the graphs of hetaerolite nano-particles are depicted in Figure 3. Hetaerolite nano-particles XPS spectrum exhibited peaks at 530.6, 642.6, 654.2, 1022.1 and 1045.1 eV which are responsible for O 1 s, Mn 2p_3/2_, Mn 2p_1/2_, Zn 2p_3/2_ and Zn 2p_1/2_ peaks, respectively. The spin–orbit splitting between Mn 2p_3/2_ and Mn 2p_1/2_ is 11.6 eV while 23.0 eV is observed among Zn 2p_3/2_ and Zn 2p_1/2_. These results are comparable to the values report earliar [18], [22]. The above results are consisted with XRD which confirms the synthesis of ZnMn_2_O_4_.

**Figure 4:**
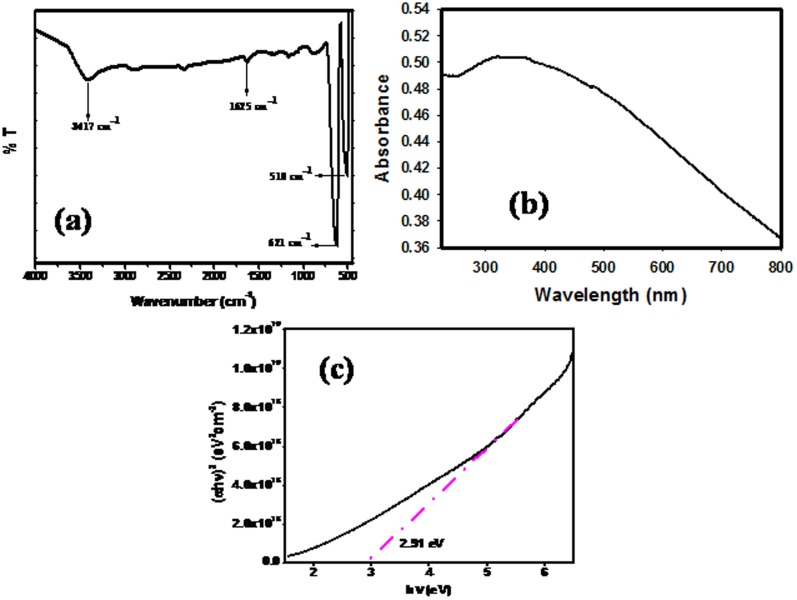
Typical FT-IR (a), UV-vis (b) spectrum and (ahυ)^2^ vs hυ graph (c) of the synthesized hetaerolite nano-particles.

The chemical structure of the hetaerolite nano-particles was also examined by FTIR analysis which was recorded in the range of 400∼4000 cm^−1^ and shown in Figure 4 (a). The very intense bands observed at 510 and 621 cm^−1^ were attributed to M–O (M = Zn, Mn) and M-O-M bonds, respectively. Supplementary peaks centered at 3417 and 1625 cm^−1^ were assigned to H_2_O absorbed from the environment [23].

The photocatalytic performance of a photocatalyst can be evaluated by its optical properties, one of the essential requirements for a photocatalyst. Thus optical properties of hetaerolite nano-particles were scrutinized by using a UV-Vis. spectrophotometer and the spectrum is shown in Figure 4 (b). In UV/visible absorption method, energy band gap of nanomaterials can be acquired by analyzing their optical absorption. UV–vis absorption spectra exhibited a broad absorption peak and showed band gap energy equal to 2.91 eV which is calculated by Tauc's formula. The relation between absorption coefficient (α) and the incident photon energy (hυ) is given by the equation [24].
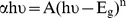
where A is a constant, E_g_ is the band gap of the material and the exponent n depends on the type of transition, n = 1/2, 2, 3/2 and 3 corresponding to allowed direct, allowed indirect, forbidden direct and forbidden indirect, respectively. Taking n = 1/2, we have calculated the direct energy band gap from the (αhυ)^1/n^ vs. hυ plots (Figure 4 (c).

### 2. Chemical sensing properties

**Figure 5:**
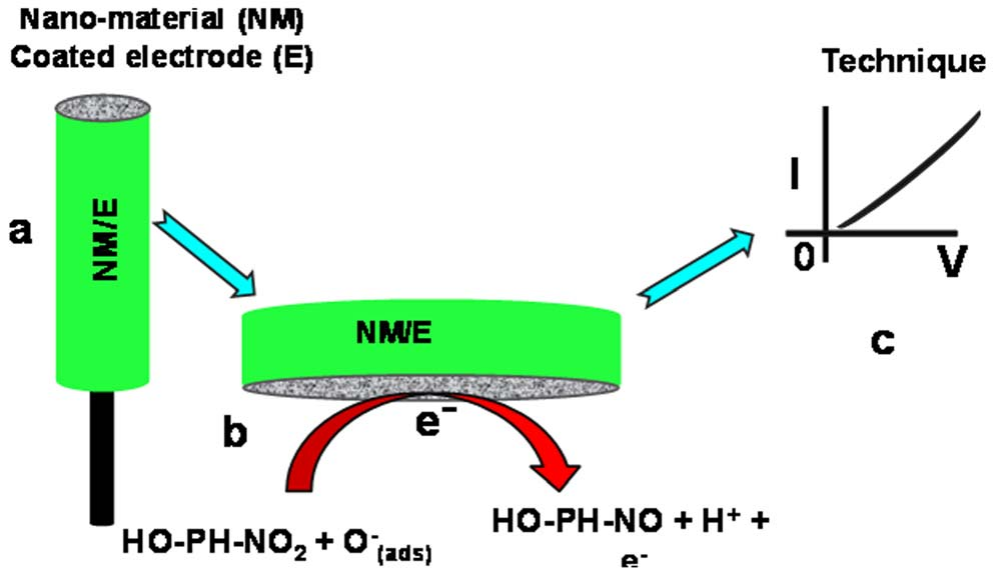
Schematic views of (a) fabricated AgE with hetaerolite nanoparticles and conducting binders, (b) reaction occurred at fabricated AgE (c) I–V detection technique.

**Figure 6:**
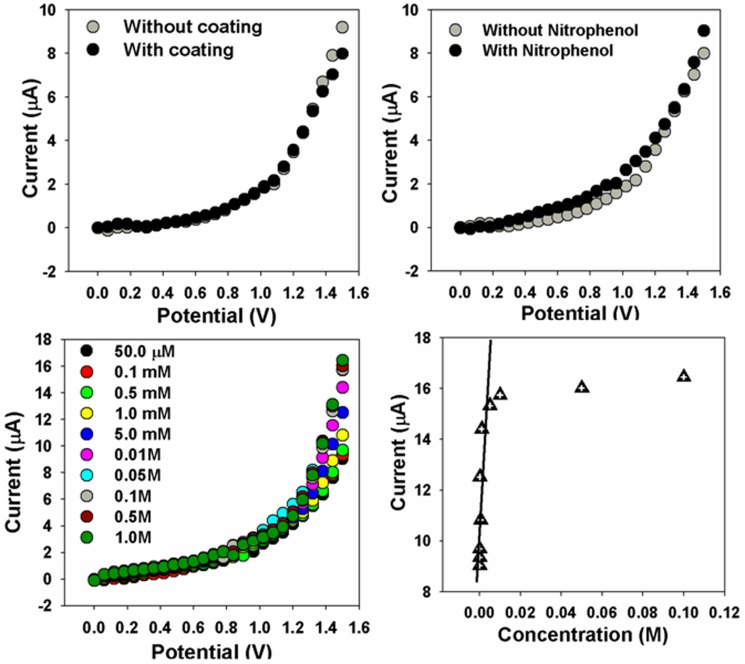
I–V characterization of hetaerolite nano-particles (a) Comparison of with and without hetaerolite nano-particles coating on GCE, (b) Comparison of with and without *p*-nitrophenol injection, (c) Concentration variation of *p*-nitrophenol, and (d) calibration plot.

The hetaerolite nano-particles were utilized for the recognition of *p*-nitrophenol in aqueous media in order to study their chemical sensing properties toward *p*-nitrophenol [25], [26]. I–V technique was used to measure the electrical response hetaerolite nano-particles sensor for *p*-nitrophenol which is shown in Figure 5 and Figure 6.

I–V curves for the glassy carbon electrode without coating (without hetaerolite nano-particles) and after coating (with hetaerolite nano-particles) were measured and shown in Figure 6 (a). The hetaerolite coated glassy carbon electrode (black square) illustrates not as much current response in contrast to the naked glassy carbon electrode (gray square), which might be attributed to resistance originated by hetaerolite nano-particles along with binders coated on the electrode surface [27].

Figure 4 (b) illustrates electrical reactions of the hetaerolite nano-particles without *p*-nitrophenol (gray-dotted line) and with 100.0 µL *p*-nitrophenol (dark-dotted line) in 0.1 M phosphate buffer solution (pH = 7.0). It is observed from the Figure 6 (b) that by adding the target chemical, hetaerolite nano-particles demonstrate a noteworthy enhancement in electrical current that reveals the sensitivity of hetaerolite nano-particles to *p*-nitrophenol. Consequently by injection of analyte, the augmentation in electrical response shows that hetaerolite nano-particles exhibit a fast and sensitive reply to the target chemical which might be due to speedy redox reaction (electron exchange) and fine electro-catalytic oxidation properties of the hetaerolite sensor. *p*-Nitrosophenol generally undergo reduction and produces *p*-hydroxylaminophenol. In second step, oxidation of *p*-hydroxylaminophenol takes place which give rise to 4-nitrosophenol and the subsequent reversible reduction.

The influence of *p*-nitrophenol concentration on the electrical reaction of hetaerolite nano-particles was examined by consecutive addition of *p*-nitrophenol in the range of 50.0 µM to 1.0 M into 0.1 M PBS solution (pH = 7.0) and the graph is portrayed in Figure 6 (c). Enhancement of the electrical current with rising *p*-nitrophenol concentration is observed which designates that the hetaerolite nano-particles conductivity was enhanced by the increase in the concentration of the target chemical [28]. The mechanism of *p*-nitrophenol sensing is graphically shown in Figure 5.

Calibration curve (Figure 6 (d)) was plotted from the difference in target concentration. The calibration curve portrays two sensitivity areas; the region at inferior concentrations (physisorption process) is linear up to 0.005 M with correlation coefficient (R) of 0.7599. The sensitivity is determined from the slope of the lesser concentration section of calibration curve, which is 1.500 µA.cm^−2^.mM^−1^. The linear dynamic range reveals from 50.0 µM to 0.05 M and the detection limit was estimated, based on signal to noise ratio (S/N), to be 20.0 µM. Above 0.005 M concentration the sensor become saturated due to chemisorption method which could be due to the lack of free hetaerolite nano-particles sites for *p*-nitrophenol adsorption [28].

### 3. Photocatalysis

#### 3.1. Solar photocatalytic performance of hetaerolite nanoparticles

The solar photo-catalytic performance of hetaerolite nanoparticles was evaluated by degrading brilliant cresyl blue under solar light [29]. In this study two sets of photo-catalytic reaction were carried out utilizing hetaerolite nanoparticles. Irradiation of brilliant cresyl blue under visible light degraded a little amount of dye at pH 7 without the catalyst (hetaerolite nano-particles) indicating a photolysis reaction. Photo-catalytical degradation of brilliant cresyl blue solution was carried out in the presence of hetaerolite nano-particles under visible light irradiation at different pHs and the effects of pH on the photo-catalytic degradation of brilliant cresyl blue studied under solar light irradiation [6]. Hetaerolite nanoparticles showed efficient activity for degradation of brilliant cresyl blue at different pH under solar light irradiation.

**Figure 7:**
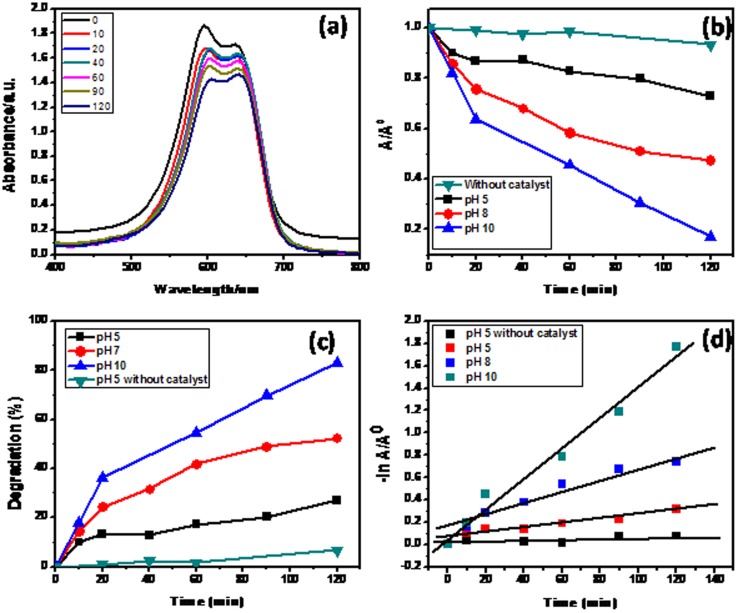
Typical plot for (a) Change in the absorption spectrum of brilliant cresyl blue (b) Change in absorbance vs irradiation time for brilliant cresyl blue at different pH, (c) % degradation vs irradiation time and (d) reaction kinetic for brilliant cresyl blue at different pH in the presence of hetaerolite nanoparticles.

An aqueous suspension of brilliant cresyl blue was irradiated with solar light in the presence of hetaerolite nanoparticles and this led to the alteration of absorbance with irradiation time [8]. Figure 7 (a) display transformation in absorption for the photo-catalytic degradation of brilliant cresyl blue at different time intervals which showed decline in absorption strength. It is clear that the absorbance at 595 nm steadily reduces with increase in irradiation time. Figure 7 (b) illustrates the transformation in absorbance with change in irradiation time for the brilliant cresyl blue in the presence and absence of hetaerolite nano-particles. Irradiation of brilliant cresyl blue solution in the presence of hetaerolite nano-particles leads to decline in absorption intensity. Figure 7 (c) demonstrates % degradation of brilliant cresyl blue in the presence and absence of hetaerolite nano-particles with respect to irradiation time (min). Degradation (%) graph shows that 27%, 52% and 83% of brilliant cresyl blue is degraded at pH 5, 8 and 10, correspondingly in the presence of hetaerolite nano-particles after 120 minutes of irradiation time while in the absence of hetaerolite nano-particles, no apparent loss of brilliant cresyl blue might be seen.

Hetaerolite nanoparticles were further compared with TiO_2_ which is a dynamic photo-catalyst and displayed an imperative role in the detoxification of polutants [9]. However TiO_2_ only promotes photo-catalysis upon irradiation by UV light because it absorbs only in the UV region. Hetaerolite nanoparticles promote photo-catalysis by irradiation with visible light because the solar spectrum consists of 46% visible light while the UV light is only 5–7% in the solar spectrum. Thus it is vital to grow an active photo-catalyst which can promote photo-catalysis in the visible region.

#### 3.2. Effect of pH

The influence of pH on the visible light photocatalytic degradation of brilliant cresyl blue was examined in pH range 5–10 and compared with the brilliant cresyl blue degradation at various pH in the presence of hetaerolite nano-particles (Figure 7). It exhibited the same trend of degradation at all pH but exhibited high photo-catalytic degradation at pH 10 as compared to pH 5 and 8. The results showed that the rate of decomposition of brilliant cresyl blue increases with increase in pH and at pH 10, brilliant cresyl blue was degraded 83% in the presence of hetaerolite nano-particles. The photocatalytic performance of hetaerolite nano-particles was attributed to the surface electrical properties, which facilitate the brilliant cresyl blue adsorption. It is beneficial for the promotion of a visible light generated charge carrier i.e. electron to the surface which leads to formation of hydroxide radical. Thus high pH makes the surface of hetaerolite nano-particles negatively charged and also supports the formation of OH^−^ radicals [30]. Both these factors favor the attraction and degradation of brilliant cresyl blue. It is clear from the results that pH has a substantial influence on the photocatalytic degradation of brilliant cresyl blue and hetaerolite nano-particles synthesized by a very straightforward synthesis process exhibit considerable solar photo-catalytic activity towards brilliant cresyl blue degradation. This demonstrates the applicability of the solar photo-catalyst towards organic pollutants and the potential to extend the applications to environmental pollutants.

#### 3.3. Reaction kinetics of photo-degradation

In order to realize the degradation behavior, we studied the degradation pattern of brilliant cresyl blue by Langmuir–Hinshelwood (L–H) model. Langmuir–Hinshelwood (L–H) model well defines the relationship among the rate of degradation and the preliminary concentration of brilliant cresyl blue in photo-catalytic reaction [31]. The rate of photo-degradation was calculated by using Equation 1:
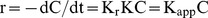
(1)Where r represents the degradation rate of brilliant cresyl blue, *K*_r_ is the reaction rate constant, K is the equilibrium constant, C is the reactant concentration. When C is very diminutive, then KC is insignificant and Equation 1 turns into first order kinetic. Consider preliminary conditions (t = 0, C = C_0_), Equation 1 turn into Equation 2.
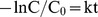
(2)Half-life, t_1/2_ (in min) is
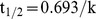
(3)
Figure 7 (d) demonstrated degradation of brilliant cresyl blue which pursued first-order kinetics (plots of ln(C/C_0_) vs time showed linear relationship). First-order rate constants, calculated from the slopes of the ln(C/C_0_) vs time plots and the half-life of the degraded brilliant cresyl blue can simply determined by Equation 3 [30], [31]. Rate constant for hetaerolite nano-particles were found to be 0.000526 min^−1^ (t_1/2_ = 1317.5 min), 0.00212 min^−1^ (t_1/2_ = 326.9 min), 0.00596 min^−1^ (t_1/2_ = 116.3 min) and 0.0136 min^−1^ (t_1/2_ = 51.0 min). Thus the kinetic study revealed that hetaerolite nano-particles is a proficient photo-catalyst for degradation of organic noxious wastes.

#### 3.4. Mechanism of photo-degradation

**Figure 8:**
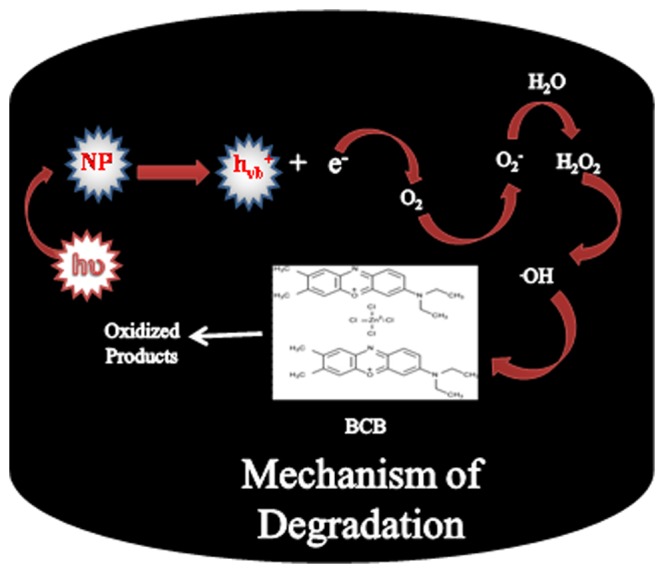
Mechanism of photodegradation of brilliant cresyl blue in the presence of hetaerolite nano-particles.

In the current study, a heterogeneous photo-catalysis method was employed for the degradation of brilliant cresyl blue. Briefly, when hetaerolite nano-particles were exposed to light having energy the same or superior to the band gap of nanoparticles, the development of an electron and hole pair occurs on the surface of hetaerolite nano-particles. If charge partition is sustained, subsequently the electron hole pair reacts with brilliant cresyl blue in the presence of oxygen. Hydroxyl radicals (OH^•^) and superoxide radical anions (O_2_^•−^) are assumed to be the major degrading mediators (oxidizing species) and oxidative reactions result in the degrading (oxidation) of the brilliant cresyl blue. The whole mechanism of photo-activity of hetaerolite nano-particles is depicted in Figure 8 [30], [31].

### Conclusion

Well-crystalline body-centered tetragonal hetaerolite nano-particles based *p*-nitrophenol electrochemical sensor has been fabricated. Hetaerolite nano-particles were produced by a hydrothermal process. The featured structural characterizations proved that the manufactured hetaerolite is optically active, well-crystalline, body-centered tetragonal nanoparticles. Sensing toward *p*-nitrophenol was executed which showed excellent sensitivity and limit of detection with quick response time. Moreover the solar photo-catalytic property of hetaerolite nanoparticles was utilized in the degradation of brilliant cresyl blue. Thus it is concluded that the hetaerolite nanoparticles are an attractive sensor material and active photo-catalyst for accomplishing a proficient chemical sensor and photo-catalyst for application within water resources and health monitoring.
